# Peri-implant phenotype, calprotectin and MMP-8 levels in cases diagnosed with peri-implant disease

**DOI:** 10.1007/s00784-024-05798-w

**Published:** 2024-06-28

**Authors:** Yasemin Beliz Önder, Nazli Zeynep Alpaslan

**Affiliations:** 1 Faculty of Dentistry, Department of Periodontology, Bilecik Şeyh Edebali University, Bilecik, Turkey; 2 Faculty of Dentistry, Department of Periodontology, Van Yuzuncu Yil University, Van, Turkey

**Keywords:** Calprotectin, MMP-8, Peri-implantitis, Phenotype

## Abstract

**Objectives:**

The purpose of this prospective cohort study is to evaluate the effect of peri-implant phenotype (PPh) on the severity of peri-implant diseases and the results of non-surgical mechanical treatment (NSMT), along with calprotectin (CLP) and MMP-8(matrix metalloproteinase-8) levels.

**Materials and methods:**

77 implants from 39 patients were included. The implants were categorized Group-1(peri-implant mucositis), Group-2(peri-implantitis).Baseline (0. Month-PrT) clinical parameters (PD, GI, PI, BOP, CAL) and radiographic bone loss were documented, and peri-implant crevicular fluid (PICF) samples were collected. Various intruments and methodologies were employed to assess PPh components (mucosa thickness, supracrestal tissue height, keratinized mucosa) and peri-implant attached mucosa (AM). NSMT was applied to diseased implant sites. All clinical parameters were reassessed again by taking PICF samples at the 6th month-after treatment (PT). In PICF samples obtained from both groups, MMP-8 and CLP levels were evaluated using the ELISA test.

**Results:**

PrT-PD,PrT-GI,PrT-CAL and PrT-BOP percentage values in Group-2 were significantly higher than Group-1.PrT-PD,PrTPI scores are significantly higher in thin biotype implants. All components of the PPh and AM were significantly lower in thin biotype. Intra-group time-dependent changes of MMP-8 and CLP were significant in both groups (*p* < 0.05). When the relationship between thin and thick biotype and biochemical parameters was evaluated, the change in PrT-PT didn’t show a significant difference (*p* > 0.05).

**Conclusions:**

PPh plays a role in influencing the severity of peri-implant diseases. However, the impact of phenotype on NSMT outcomes was similar in both groups.

**Clinical relevance:**

The PPh should be considered when planning implant surgery.

## Introduction

In contemporary dentistry, the restoration of both the form and function of missing teeth holds significant importance. The term ‘dental implant,’ coined to address these needs, refers to tissue-compatible devices placed within or on the jawbone [1].

The soft tissue that forms around dental implants is referred to as ‘peri-implant mucosa’ [2]. Despite successful osseointegration, inflammation in the peri-implant mucosa can impact implant survival and lead to soft and/or hard tissue loss [3]. With the widespread use of dental implants, there has been a notable increase in both complications and peri-implant diseases [4]. In 2018, a workshop report included conditions and diseases related to implants in the classification for the first time, defining peri-implant health to encompass peri-implant mucositis, peri-implantitis, and peri-implant soft and hard tissue deficiencies [5]. Peri-implant health is characterized by the absence of clinical signs of inflammation, such as bleeding, edema, suppuration, swelling, contour disorder, and erythema in the peri-implant mucosa. Researchers argue that probing depth (PD) alone is insufficient and emphasize that peri-implant health can be present even in implants with reduced bone support [6].

Peri-implant mucositis is defined as an inflammatory condition with clinical inflammation symptoms observed in the peri-implant mucosa, sometimes accompanied by pain [7]. The progression of this inflammatory condition to the bone surrounding the implant, causing destruction, leads to peri-implantitis [3]. Another implant-related condition is peri-implant soft and hard tissue deficiencies, which may develop before or after implant surgery depending on various factors [8].

The primary etiological factor in the development of peri-implant diseases is dysbiotic oral microbiota [9]. Additionally, prosthetically poorly positioned implants, errors during prosthetic restoration cementation, iatrogenic factors [10], prosthesis duration in function, implant design, age, systemic disorders (such as diabetes), smoking [11], radiation therapy, failure to include supportive treatment, implant materials, and local and systemic conditions like surface characteristics, prosthesis design, and peri-implant tissue phenotype (PPh) are prominent in the etiology of peri-implant diseases [2]. The impact of PPh on implant health and aesthetics has been at the center of significant debate in the last decade [12]. PPh includes keratinized tissue width, mucosa thickness and supracrestal tissue height (STH) components. It has been confirmed by various studies that all these components are effective on implant health [13, 14].

Various methods and techniques have been developed for diagnosing peri-implant health and diseases. In recent years, there has been a focus on reaching a diagnosis through PICF (peri-implant crevicular fluid) analysis, providing an objective evaluation [15]. Studies [15, 16] have indicated that several biomarkers are elevated in PICF obtained from diseased implant sites compared to healthy peri-implant tissues. Matrix metalloproteinase-8 (MMP-8), a collagenase, is recognized as a key biomarker used for predicting, diagnosing, prognosticating treatment, and classifying periodontal disease. Calprotectin (CLP), identified as a biomarker of bone resorption, was extracted from PICF and verified to exhibit a positive correlation with both probing depth (PD) and gingival index (GI) scores [17]. Nevertheless, the existing literature linking it to peri-implant diseases is notably inadequate. In light of these revealing findings, our study aims to investigate the impact of PPh on the severity of peri-implant diseases and its role in influencing outcomes of non-surgical mechanical treatments, considering both clinical and biochemical parameters. The hypothesis of this study is that the severity of peri-implant diseases will increase in patients with a thin phenotype and the response to treatment will be less successful than in individuals with a thick phenotype.

## Materials and methods

### Study design, ethical approval, and setting

This prospective cohort study received approval from the Van YYU (Yüzüncü Yıl University) Faculty of Medicine Interventional Research Ethics Committee under the reference number 17.06.2020/13. The research adheres to the principles outlined in the 1975 Helsinki Declaration, revised in 2013. The clinical trial is registered in the ClinicalTrials.gov database (NCT06173739). The research, prepared in accordance with the Strengthening the reporting of cohort studies in surgery (STROCSS) guideline [18], involved a total of 77 implants in 39 patients, comprising 21 women and 18 men aged between 18 and 65. These patients, referred to the Van YYU Faculty of Dentistry Department of Periodontology between August 2020 and March 2022 with complaints of peri-implant area inflammation and diagnosed with peri-implant disease, were included in the study. Although the study commenced with 44 patients with 83 implants, three patients were excluded due to non-compliance with inclusion criteria, one patient’s inability to maintain oral hygiene, and another patient’s failure to attend follow-up visits. Informed consent was obtained from all participants.

### Eligibility criteria and recruitment

The general inclusion criteria for participants in this study were as follows: volunteers aged between 18 and 65, absence of any systemic disease, non-smoking status, non-pregnant or breastfeeding, and no use of antibiotics or any medical treatment in the last 6 months. The study specifically focused on bone level implants of the same brand (Nucleoss ^®^ T6 Torq Gr4 surface, Izmir/Turkey), characterized by angle-free, convex profile, single-member, cemented crowns with prosthetic superstructure and no surgical procedures (hard tissue graft, free gingival graft, connective tissue graft) during implantation. Exclusion criteria included individuals meeting the definition of peri-implant health, those requiring surgical treatment for peri-implantitis, and those refusing participation in the study.

Inclusion criteria for patients with peri-implant mucositis (Group 1): There is no supporting or marginal bone loss (except remodelling) in patients diagnosed with peri-implant mucositis. There is edema, swelling, bleeding, hyperemia or suppuration in the peri-implant mucosa [19].

Inclusion criteria for patients with peri-implantitis (Group 2): Patients with radiographic bone loss around the implant, increased probing depth, and bleeding, edema, erythema, and hyperemia were included in this group. In addition to these, the patient was diagnosed with peri-implantitis; It depends on the radiographic bone loss being greater than the approximately 1 mm loss observed in the first year and the accompanying inflammation markers [5].

### Collection and analysis of PICF (peri-implant crevicular fluid) samples

**Fig. 1:**
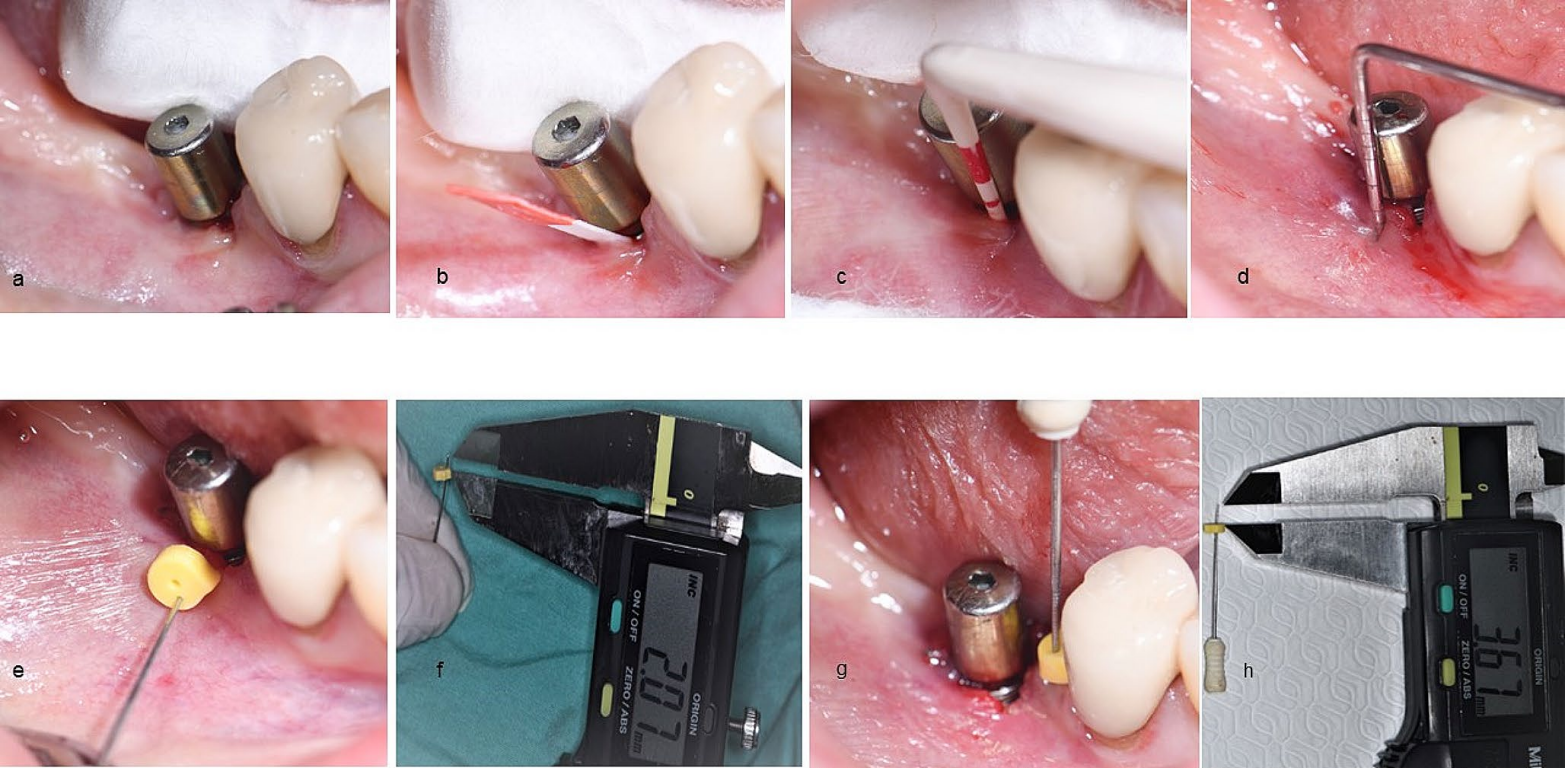
**(a)** The implant diagnosed with peri-implant disease and crown removed **(b)** PICF collection method using Periopaper **(c)** Recording clinical parameters with a plastic probe **(d)** Peri-implant attached mucosa (AM) measurement **(e)** MT determination with transgingival probing **(f)** Measuring the value of MT on the spreader with a digital caliper **(g)** STH determination **(h)** Measuring the value of STH on the spreader with a digital caliper

PICF samples were gathered on the day following crown removal to prevent potential contamination of paper strips due to the destruction and bleeding caused during the crown removal process [20]. During specimen collection, the peri-implant mucosa was air-dried using a water spray. Utilizing the in-groove method and a press without pressure, paper strips (PerioPaper^®^, Oraflow, NY, USA) (Fig. 1-b) were carefully placed into the pocket. After 30 s in the pocket, the paper strips were then transferred to Eppendorf tubes (SealRite 1.5 mL Microcentrifuge Tubes; Scientific Inc., Orlando, FL, USA) containing 500 µl of PBS (phosphate-buffered saline, pH: 7.0) and stored at -40 °C until the day of analysis.

Two PICF-impregnated paper strips were acquired from each implant diagnosed with peri-implant disease. Upon reaching the targeted number, an Enzyme-Linked ImmunoSorbent Assay (ELISA) technique was employed to assess MMP-8 and CLP levels in PICF following the manufacturer’s recommendations (Human Matrix-Metalloproteinase 8; Neutrophil collagenase ELISA kit and Human Calprotectin ELISA kit, Jiaxing, Zhejiang, Korea). Absorbance values were measured using an ELISA reader (µQuant™ ELISA Microplate Reader, BioTek^®^ Instruments, Inc., VT, USA) at a wavelength of 450 nm.

### Clinical and radiographic measurements

Following the removal of crowns, demographic information (gender, age, education level, tooth brushing frequency, interface care) and clinical measurements were obtained and documented from eligible patients who met the inclusion criteria and completed the examination. All the data collected by same researcher (Onder YB). To ensure intra-investigator calibration, the investigator conducted a double evaluation on a minimum of 30 non-trial implants. Assessments were consistently conducted on the same patients at intervals of at least 60 min. The inter-class correlation coefficient for the investigator was found to range from 0.89 to 0.97, indicating a high level of agreement between repeated measurements. Clinical parameters, including probing depth (PD), plaque index (PI) [21], gingival index (GI) [21], bleeding on probing (BOP) [22] and clinical attachment level (CAL) [23], were recorded using a single type of plastic probe (KERR- Hawe Caliber Plastic Periodontal Probe, Switzerland) (Fig. 1-c). Scores were documented on a specially prepared index form. Radiographic assesment of bone levels in the mesial and distal regions on panoramic radiographs on the date of implant placement and the date examined was performed using NIH ImageJ software [24], accessible at https://imagej.nih.gov/ij/docs/guide/. Radiographic bone loss data were not measured again at 6 months because the patients did not undergo any surgical intervention.

### Peri-implant soft tissue phenotype (PPh)

In the first session, in which all 4 components of PPh were examined, firstly; the measurement of peri-implant keratinized tissue width (KT) involved using a probe, positioning it at the mid-buccal of the abutment as a reference point. Following the determination of the mucogingival junction through the rolling technique [25] the probe was maintained parallel to the long axis of the implant and inserted between the marginal gingiva and the mucogingival line (Fig. 1-d). Additionally, peri-implant attached mucosa (AM) was computed by subtracting the probing depth from the KT width value.

Mucosal thickness (MT) was determined through the transgingival probing measurement technique [26] (Fig. 1-e). In this procedure, local anesthesia (Ultracain D-S Forte 40 mg/mL + 12 mcg/Ml, Kırklareli / Turkey) was administered to the implant area for measurement. A spreader (no:15) (G-Star Medical, Guang-dong, China) was then applied perpendicularly to the keratinized mucosa in the mid-buccal area. The spreader was advanced until contact was made with the bone or implant surface. The position of the spreader was checked occlusally with a mouth mirror, ensuring contact between the stopper and the gingiva. Once bone and implant contact were confirmed, a stopper marked the point, and the spreader was removed without damaging the stop point. The distance determined by the spreader was precisely measured using a digital caliper (Mitutoyo Corporation, Kanagawa, Japan) with 0.01 mm calibration (Fig. 1-f) [27]. Measurements were taken from two areas (mesial and distal) in the coronal part of the KT near the abutment and from two areas (mesial and distal) in the apical part close to the mucogingival junction, and their averages were calculated. Similar to the MT measurement technique, transgingival probing was conducted with spreader (no:15), but it was placed parallel to the long axis of the implant for STH (Supracrestal Tissue Height). The bone contact point was marked with a stopper and measured with a digital caliper (Mitutoyo Corporation, Kanagawa, Japan). Measurements were taken from four regions (mesial, distal, lingual/palatinal, and buccal), and the STH was determined by averaging the values (Fig. 1-g, h).

**Fig. 2:**
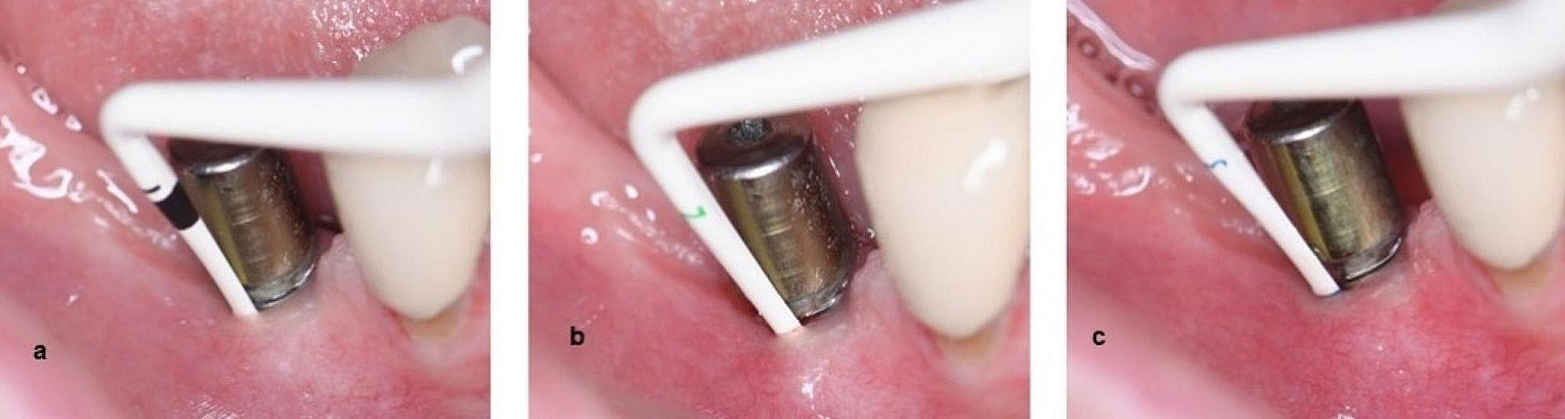
Biotype measurement with colour coded probe **(a)** White **(b)** Green **(c)** Blue

For determining the biotype, color-coded biotype probes (Hu-Friedy^®^ Colorvue Biotype Probe/ PBTPKIT12) were utilized [28]. Probes with white, green, and blue tips were inserted into the mid-buccal gingival groove of the implant surface. The white probe tip was initially placed in the peri-implant groove, and if reflected from the margin, the other tips were not moved, indicating a thin biotype. If the white tip did not reflect from the margin, the green tip was used. If reflected from the margin, it was recorded as a middle biotype, and the process did not proceed to the blue tip. Finally, if the green tip did not reflect from the margin, the blue tip was placed in the groove, and if reflected, it was considered a thick biotype. If no reflection occurred, the peri-implant soft tissue was categorized as a very thick biotype (Fig. 2).

### Non-surgical mechanical treatment (NSMT)

In the second session, NSMT was administered to patients who were summoned to the clinic after the collection of PICF samples and determination of the soft tissue phenotype. During the phenotype determination process, NSMT was gently applied to patients who were already under local anesthesia by the same clinician (Önder YB) until it was discerned that the surfaces were adequately debrided. In this procedure, a titanium curette (Hu-friedy^®^ 1–2 minifive™ IMPM1/2T, Chicago, AB) was used and subgingival irrigation performed with physiological saline [29] on the affected implant surfaces. No specific medication was prescribed. Post-treatment, oral hygiene instructions were provided to the patients, and they were scheduled for follow-up appointments at the 1st, 3rd, and 6th months, during which they were included in supportive periodontal treatment. Patients summoned to the clinic in the first month were referred to the Van YYU Department of Prosthodontics for a temporary prosthesis. If improvements were observed by the 6th month, consultation with the relevant department was sought for consideration of permanent prosthetic restoration.

### Statistical analysis

The sample size of the study was calculated with PASS (Power Analysis and Sample Size Software (2017) NCSS, LLC. Kaysville, Utah, USA the program ncss.com/software/pass). Based on the results of a similar study previously conducted [15], MMP-8, CLP of peri-implantitis and peri-implant mucositis groups and taking into account the ratios in the comparison of clinical parameters, bidirectional alternative hypothesis, with 80% power and 5% Type I error margin, with a sample size of at least n:77.

To assess the impact of PPh on the severity and recovery of peri-implant diseases, a study was conducted involving a minimum of 77 implants. The study aimed for a power value of 80%, with a margin of error set at 5%, a confidence level of 95%, and an effect size of 0.66. Statistical analyses were performed using Shapiro-Wilk’s and/or Kolmogorov-Smirnov’s tests for variables exhibiting a normal distribution. In cases where variables deviated from a normal distribution, the Mann-Whitney U Test and Kruskal-Wallis H were employed for group comparisons, while Chi-Square tests were utilized for assessing intergroup relations. The Wilcoxon Test was applied to variables that did not conform to a normal distribution. A significance level of *p* < 0.05 was considered statistically significant in all analyses.

## Results

This study was initially designed to include 44 patients, however, it was completed with a total of 39 patients and 77 implants. During the course of the study, 5 patients were lost to follow-up: 3 due to radiographic bone loss exceeding 6 mm, 1 due to inability to maintain oral hygiene, and 1 due to non-attendance at the 6-month follow-up sessions, hence they were excluded from the analysis.

Based on the findings derived from the data of patients in Group-1 (peri-implant mucositis) and Group-2 (peri-implantitis), there is no statistically significant distinction observed in terms of gender, interface care, age, and education level across the diagnosis groups. However, a statistically significant difference is evident between the groups concerning tooth brushing frequency (TBF) (*p* = 0,002). The count of patients who brush their teeth twice a day is notably higher in Group-1 compared to the Group-2.

### Clinical parameters

Group-2 exhibits significantly higher values for PD, GI, CAL, and BOP percentages compared to Group-1 (*p* < 0.05), while no significant difference is observed between the groups in terms of PI values (*p* > 0.05).

Radiographic bone loss is evident in all patients in Group-2, and a statistically significant difference is noted between the groups regarding extent of radiographic bone loss (*p* = 0.001).

Pre-treatment probing depth (PrT-PD), PrT-GI, PrT-CAL and PrT-BOP percentage values in Group-1 are statistically significantly lower than in Group-2 (*p* < 0.05). Additionally, post-treatment probing depth (PT-PD) and PT clinical attachment level (PT-CAL) are significantly lower in Group-1 compared to Group-2 (*p* < 0.05). Moreover, there is no statistically significant difference between the groups with respect to PT-GI, PrT-PI, PT-PI, and PT-BOP (*p* > 0.05). In addition, the decreases in all clinical parameters except PI over the 6-month period were found to be significantly higher in the peri-implantitis group. The variations in all clinical parameters over time are detailed in Table 1.

**Table 1:** Comparison of intra- and inter-group parameters

		Group-1	Group-2	Total	p**	z	pΔ
		Mean ± SD	Mean ± SD	Mean ± SD			
**PD (mm)**	PrT	2.47 ± 0.93	4.63 ± 0.96		**0.001**		
	PT	2.08 ± 0.75	3.2 ± 1.1		**0.001**		
	z	-3.462	-5581				
	p*	**0.001**	**0.001**				
	Difference (PrT-PT)	-0.39 ± 0.61	-1.43 ± 0.84	-1 ± 0.91		-5.213	**0.001**
**GI (s)**	PrT	1.53 ± 0.55	1.9 ± 0.48		**0.002**		
	PT	0.98 ± 0.62	0.88 ± 0.66		0.468		
	z	-3.639	-5.441				
	p*	**0.001**	**0.001**				
	Difference (PrT-PT)	-0.55 ± 0.66	-1.02 ± 0.69	-0.82 ± 0.71		-2.812	**0.005**
**PI (s)**	PrT	1.25 ± 0.88	1.27 ± 0.77		0.933		
	PT	0.73 ± 0.89	0.54 ± 0.66		0.473		
	z	-2.674	-4.356				
	p*	**0.007**	**0.001**				
	Difference (PrT-PT)	-0.52 ± 1.06	-0.73 ± 0.88	-0.64 ± 0.96		-1.192	0.233
**CAL (mm)**	PrT	3.27 ± 1.05	5.53 ± 0.73		**0.001**		
	PT	2.94 ± 1.08	4.22 ± 1.4		**0.001**		
	z	-1.562	-4.906				
	p*	0.118	**0.001**				
	Difference (PrT-PT)	-0.33 ± 1.09	-1.31 ± 1.22	-0.9 ± 1.26		-3.387	**0.001**
**BOP (%)**	PrT	66.03 ± 35.16	84.73 ± 25.66		**0.001**		
	PT	23.38 ± 31.61	19.89 ± 28.93		0.606		
	z	-3.806	-5.533				
	p*	**0.001**	**0,001**				
	Difference (PrT-PT)	-42.66 ± 44.67	-64.84 ± 34.69	-55.62 ± 40.39		-2.245	**0.025**
**R.Bone Loss (mm**)	PrT	0.28 ± 0.89	3.29 ± 2.59		**0.001**	**-**6.96	
**CLP (ng)**	PrT	107978.9 ± 26.647	116923.2 ± 15676.05		0.126		
	PT	93680.06 ± 27461.93	93.896 ± 27455.37		0.1		
	z	-2.842	-4,713				
	p*	**0.004**	**0.001**				
	Difference (PrT-PT)	-14298.9 ± 24444.01	-23027.2 ± 26727.6	-19399.8 ± 26000.9		-1.323	0.186
**MMP-8 (ng)**	PrT	3.64 ± 1.09	4.04 ± 0.88		0.142		
	PT	2.71 ± 1.05	3.11 ± 1.15		0.179		
	z	-3.908	-4.825				
	p*	**0.001**	**0.001**				
	Difference (PrT-PT)	-0.93 ± 1.11	-0.92 ± 0.95	-0.93 ± 1.01		-0.331	0.741

*Wilcoxon test **Δ Mann Whitney U test z: coefficient of variation; *PrT* Pre-Treatment, *PT* Post-Treatment, *CLP* calprotectin, *MMP-8* Matrix metalloproteinase-8, *ng* nanogram, *PD* probing depth, *GI* gingival index, *PI* plaque index, *CAL* clinical attachment level, *BOP* bleeding on probing, *mm* milimeter, *s* scor, *SD* standard deviation, p*:intergroup, p**:intragroup, *p*Δ: time depended (6 months) difference, R.:radiographic, *p* < 0.05 significance level

### Evaluation of peri-implant mucosa phenotype (PPh)

A uniform distribution (*p* > 0.05) was observed between the groups concerning peri-implant attached mucosa (AM), KT, and MT. However, the STH in Group-2 was notably lower than in Group-1 (*p* = 0.027). Although not reaching statistical significance, it was noted that KT, the quantity of AM, and MT tended to be higher in Group-1 implants compared to Group-2 implants (Table 2).

**Table 2:** Evaluation of the relationship between groups with PPh

Groups	STH (mm) (Mean ± SD)	Peri-implant attached mucosa (AM) (mm) ((Mean ± SD)	KT (mm) ((Mean ± SD )	MT (mm) ((Mean ± SD)
**Group-1** **(*n***:**32)**	3.6 ± 1.12	2.37 ± 1.53	3.03 ± 1.78	2.06 ± 0.8
**Group-2** **(*n***:**45)**	3.03 ± 0.9	2.21 ± 1.23	2.56 ± 1.49	2 ± 0.85
***p *** **value**	0.027*	0.832	0.282	0.559
**Thin Biotype** **(*n***:**21)**	2.87 ± 0.76	1.4 ± 0.88	1.74 ± 1.21	1.07 ± 0.28
**Thick Biotype** **(*n***:**26)**	3.59 ± 1.27	2.85 ± 1.32	3.52 ± 1.62	2.76 ± 0.67
***p***	0.023*	0.001*	0.001*	0.001*

**p < 0.05 significance level, Mann Whitney U test, *mm* milimeter, *SD* standard deviation, *n* number

Upon scrutinizing the association between the data derived from color-coded biotype probes and the groups, no statistically significant difference was identified (*p* > 0.05). Individuals exhibiting thin, medium, thick, and very thick biotypes demonstrated a homogeneous distribution within both groups (Table 3). To further illuminate the study hypothesis, a secondary statistical analysis was conducted on the obtained data, specifically focusing on thin and thick biotypes. Implants reflecting white color from the peri-implant mucosa were categorized as thin biotype, whereas peri-implant mucosa reflecting blue color or showing no color reflection was classified as thick biotype. Among the evaluated implants, 21 were identified as having a thin biotype, while 26 were determined to possess a thick biotype. The peri-implant mucosa of 30 implants exhibited a medium biotype and was not included in this categorization (Table 2). When all parameters determining the PPh are evaluated based on thin and thick biotype; it was determined that the data for all phenotype components were significantly lower in the thin biotype than in the thick biotype (*p* < 0.05) (refer to Table 2).

**Table 3:** Intergroup distribution of results obtained from color-coded biotype probes

		Groups						Chi square test	
		Group-1		Group-2		Total			
		*n*	%	*n*	%	*n*	%	Chi-square	*p*
**Probe color reflected from the margin**	**White**	9	28.13	12	26.67	21	27.27	3.285	0.35
	**Green**	9	28.13	21	46.67	30	38.96		
	**Blue**	8	25	7	15.56	15	19.48		
	**None**	6	18.75	5	11.11	11	14.29		
	**Total**	32	100	45	100	77	100		

*p < 0.05 significance level, Chi Square Test, %: percentage

**Fig. 3:**
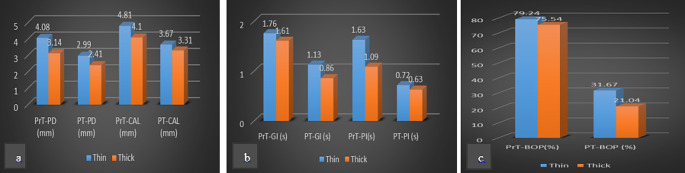
The impact of biotype on clinical parameters (**a)** PD-CAL parameters (**b) **GI and PI scors (**c) **BOP percentage

PrT-PD and PrT-PI scores were notably higher in implants with a thin biotype (n:21) compared to implants with a thick biotype (n:26) (*p* < 0.05). Although not reaching statistical significance, PrT-GI, PrTCAL, and PrT-BOP percentage values tended to be higher in implants with thin biotypes than in implants with thick biotypes (Fig. 3). However, no statistically significant results were obtained in time-dependent changes in all clinical parameters (refer to Table 4).

**Table 4:** The time-dependent change of clinical parameters with biotype values comparison

Clinical parameters	Biotype	*n*	Min	Max	SD	z	*p*
**PT-PrT PD (mm)**	**Thin**	21	-3	0.16	0.95	-1.237	0.216
	**Thick**	26	-2.67	0.5	0.88		
	**Total**	47	-3	0.5	0.92		
**PT-PrT GI (s)**	**Thin**	21	-1.66	0.33	0.63	-0.775	0.439
	**Thick**	26	-2	1	0.77		
	**Total**	47	-2	1	0.71		
**PT-PrT PI (s)**	**Thin**	21	-2	1	0.83	-1.78	0.075
	**Thick**	26	-2	1	0.89		
	**Total**	47	-2	1	0.88		
**PT-PrT CAL (mm)**	**Thin**	21	-3	0	1.06	-1.036	0.3
	**Thick**	26	-4	2	1.39		
	**Total**	47	-4	2	1.25		
**PT-PrT BOP (%)**	**Thin**	21	-100	17	39.64	-0.809	0.419
	**Thick**	26	-100	67	42.84		
	**Total**	47	-100	67	41.14		

Mann Whitney U test z: coefficient of variation; *PrT* Pre-Treatment, *PT* Post-Treatment, *PD* probing depth, *GI* gingival index, *PI* plaque index, *CAL* clinical attachment level, *BOP* bleeding on probing, *mm* milimeter, *s* scor, *SD* standart deviation, *%* percentage, *n* number of implant, *p* < 0.05 significance level

### ELISA values

In our investigation, where we assessed MMP-8 and CLP biomarkers, no statistically significant difference was observed in both values among the groups in terms of baseline values. (*p* > 0.05). Despite the lack of statistical significance, the time-dependent decrease in ELISA (ng) values of Group-2 tended to be higher than those of Group-1 for CLP biomarker (refer to Table 1). Within the thin and thick biotype groups, although the PrT-MMP-8 values of implants with thin biotypes were numerically higher than those of implants with thick biotypes, this difference did not reach statistical significance. However, PrT-CLP values were found to be significantly higher in implants with thin biotypes compared to implants with thick biotypes (p:0.041) (Table 5). Furthermore, a decrease in both biomarkers obtained from PICF was observed post-treatment, but it was not found to be statistically significant.

**Table 5:** Comparison of biochemical parameters by groups

		Thin	Thick	Total	z	p*
		Mean ± SD	Mean ± SD	Mean ± SD		
**MMP-8 (ng)**	PrT	4.02 ± 0.77	3.49 ± 1.17	3.73 ± 1.04	-1.658	0.097
	PT	3.08 ± 1.16	2.32 ± 0.86	2.66 ± 1.07	-2.354	**0.019**
	Difference (PrT-PT)	-1 ± 1.06	-1 ± 0.92	-1 ± 0.98	-0.963	0.336
**CLP (ng)**	PrT	119069.7 ± 19250.5	102372.1 ± 24050.2	109832.7 ± 23364.26	-2.044	**0.041**
	PT	96.924 ± 25686.19	79797.42 ± 29708.49	87449.72 ± 28993.24	-2.076	**0.038**
	Difference (PrT-PT)	-22.146 ± 27796.8	-22.575 ± 28918.15	-22.383 ± 28115.39	-0.021	0.983

*Mann Whitney U test; z:coefficient of variation; *PrT* Pre-Treatment; *PT* Post-Treatment, *CLP* calprotectin, *MMP-8* Matrix metalloproteinase-8, *ng* nanogram, *mm* milimeter, *s* scor, *SD* standard deviation

## Discussion

Numerous methods exist in the literature for diagnosing peri-implant diseases that have arisen with the widespread use of dental implants [15]. Among them PICF analysis combined with clinical parameters and radiographic evaluation, the obtained data display valuable objective frame of the current condition of the peri-implant disease. The biomarkers analyzed in the study include MMP-8 and CLP. MMP-8, a collagenase, has been highlighted in numerous studies as one of the early-stage mediators of the disease, playing a crucial role in disease diagnosis [29–31]. CLP, a protein associated with inflammation, was found at a higher concentration in diseased areas compared to healthy areas in the gingival crevicular fluid [17]. To the best of our knowledge, this study is the first to jointly analyze and evaluate CLP and MMP-8 biomarkers in peri-implant diseases.

While bacterial biofilm accumulation is acknowledged as the primary factor in the etiology of peri-implant diseases [32], our study considers various factors, such as prosthetic restoration, implant design and material, patient’s age, systemic diseases, smoking, radiation therapy, periodontal therapy, and PPh, which also play a significant role in the development of peri-implant diseases [2, 11]. PPh includes KT, MT and STH components [12]. In measuring both MT and STH, our study employed the transmucosal probing technique with spreader No. 15, similar to the methods used by Kaya et al. [33] in 2018 and Durrani et al. [34] in 2021. The assessment of biotype utilized current special color-coded biotype probes, allowing for the categorization of biotypes into four groups [28].

No statistically significant connection was observed between demographic information (gender, age, education level, interface care) and groups, indicating a homogeneous distribution [35, 36]. However, a significant difference was identified in tooth brushing frequency between groups (higher in Group-1 patients), aligning with a similar finding in the study by Choi and colleagues published in 2020 [37]. While there was no significant difference in interface care between the groups, it was observed that the severity of the disease decreased as care increased.

Considering the intergroup comparisons of PrT-PT measured clinical parameter values in the groups, it is an expected result that all pre-treatment (PrT) clinical parameters except PI in Group-2 are significantly higher [38] (refer to Table 1). In a study published in 2018 [39], similar to our investigation, it was emphasized that PD and BOP values were significantly higher in peri-implantitis. The fact that the difference between groups in PT-GI, PT-PI, PT-BOP values is insignificant can be explained by the observation of improvement in both groups.

The hypothesis of this study is that the severity of peri-implant diseases will increase in patients with a thin phenotype and the response to treatment will be less successful than in individuals with a thick phenotype. We comprehensively evaluated all components of the phenotype, including the use of color-coded probes for biotype assessment. The results revealed that 43.75% of implants in Group-1 exhibited a thick biotype (blue tip-none), while the rate of thick biotype (white tip) implants in Group-2 was 26.67% (Table 3). Although this difference did not reach statistical significance, a negative relationship between the severity of diseases and the biotype of the peri-implant mucosa was observed. Notably, literature review did not yield any studies utilizing color-coded biotype probes in the peri-implant mucosa, and literature lacks evidence establishing the impact of biotype on the severity of peri-implant diseases. In this study, where the peri-implant mucosa biotype was homogeneously distributed, a secondary statistical analysis showed that all pre-treatment clinical parameters had higher quantitative values in the thin biotype than in the thick biotype (Fig. 3). Based on these findings, it can be asserted that patients with a thin biotype may be more susceptible to peri-implant disease [40]. However, the change in all clinical parameters after NSMT was not statistically significant in the thin and thick biotypes. In the light of these results, our hypothesis is partially confirmed. Although biotype affected the severity of the disease in our sample group, it did not affect the treatment results (Tables 4 and 5).

**Fig. 4:**
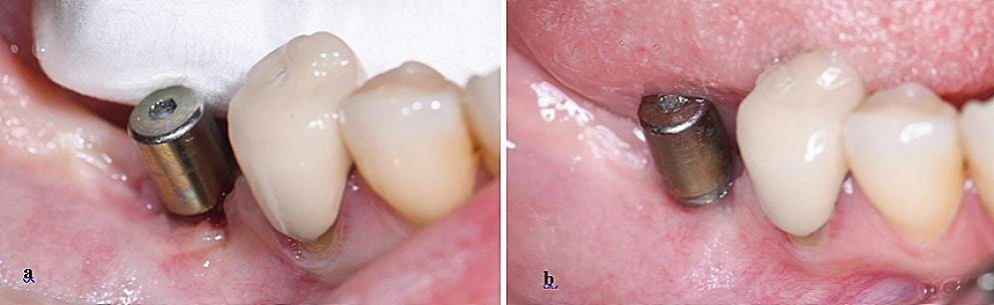
**a**. Pre-existing peri-implantitis was observed in implants with middle biotype prior to NSMT **b.** The observed improvement post-NSMT

The determination of biotype in our study involved the use of color-coded biotype probes and the measurements obtained through transgingival probing. Consequently, all values of phenotype and AM were significantly lower in implants with a thin biotype (refer to Table 2). The significant excess of STH, AM, KT, and MT in thick biotype implants is compatible with expectations. Bertl et al. [41] suggested potential inaccuracies in the evaluation of color-coded biotype probes when MT exceeds 1.5 mm. Results of the current study showed that the average MT in implants with a thin biotype is 1.07 mm, and in implants with a thick biotype the average MT was 2.76 mm. In current study color-coded biotype probes showed cases where the blue or no tip was visible at values above 1.5 mm. Therefore, this study suggests that, color-coded biotype probes can offer practical utility. The novelty of the method, instruments, and techniques employed, coupled with limited studies in this direction, may pose challenges in interpreting the findings. To provide more precise expressions and evidence-based data, we believe that conducting studies with larger sample groups using this method would be beneficial.

Considering the STH data, the substantial difference observed between the groups aligns with findings reported by Tavelli et al. [12]. In our study, precise measurements were conducted using a digital caliper and spreader, a methodology limited in the literature. In implants with thick biotypes, the average STH was 3.59 ± 1.27 mm, while in implants with thin biotypes, it was 2.87 ± 0.76 mm (refer to Table 2).

Existing literatüre [42] underscores the heightened plaque retention in cases with insufficient KT (< 2 mm), contributing to increased peri-implant disease prevalence. This research indicates an inverse relationship, demonstrating that as KT increases, the severity of peri-implant diseases decreases. Although many studies [43–45] consider cases with KT below 2 mm as insufficient, our prospective study revealed an average KT value exceeding 2 mm for both groups. Suggesting that the significance level might rise in studies with inadequate KT measurements, further research in this direction could contribute to the literature. Studies [12, 46] have demonstrated that an ample amount of AM around dental implants promotes increased hard and soft tissue stability, yielding improved aesthetic outcomes. Additionally, increased AM diminishes plaque retention [46]. Recent research [46] advocates for placing implants exclusively in areas with sufficient AM. The data obtained from this study displayed that Group-1 exhibited a higher amount of AM, although statistically insignificant. Correspondingly, Roccuzzo et al.‘s study [47] supports the notion that patients with attached gingiva and KT insufficiency are more susceptible to peri-implant diseases.

CLP, an inflammation-related protein [48], has been studied in relation to peri-implant diseases. Sakamoto et al.‘s study [17] demonstrated significantly higher CLP amounts and concentrations in diseased areas compared to healthy ones, revealing a positive correlation with clinical parameters like PD and GI scores. Kido et al.‘s research [48] yielded similar results, with higher CLP levels in diseased implants. Our study aligns with these findings, noting a higher, though not significant, PT- CLP and PrT-CLP value in Group-2 compared to Group-1. Consistent with the mentioned studies, CLP values decreased as we progressed towards health in current study. Regarding MMP-8, recognized as a key factor in peri-implantitis Arakawa et al. [49], Ziebolz et al. [50] suggested its inadequacy as a biomarker to distinguish peri-implantitis and peri-implant mucositis, reporting comparable values in both groups. Our study echoes these results, and further comprehensive studies are recommended for evidence-based outcomes. In this study, when evaluating baseline and outcome (PrT and PT) levels of biomarkers, it was observed that both biomarkers were higher in implants with a thin biotype, indicating that biotype influences the severity of peri-implant disease. Moreover, this data is statistically significant in post-treatment values. However, the 6-month change in both groups is similar. In light of this information, it would be reasonable to argue that NSMT is successful in both groups (thin-thick biotype). Biomarkers and clinical parameters have shown similar results over a 6-month period (refer to Tables 4 and 5). While biotype does not influence the outcomes of non-surgical treatment of peri-implant diseases, our hypothesis is partially validated in that it affects the severity of peri-implant diseases.

In this investigation, the spatial orientation of the dental implants within the oral cavity was not duly considered. Variations stemming from anatomical disparities and individual peculiarities, particularly within the anterior and posterior regions, as well as between the mandibular and maxillary arches, have the potential to significantly influence the outcomes of analysis. Given that statistically significant data can be obtained in studies with larger sample groups, the size of the population is considered one of the limitations of our study. Furthermore, a correlation analysis between phenotype elements and biotype was not conducted.

## Conclusion

Primary results of this study concluded that the thin and thick biotype exhibit a direct connection with peri-implant diseases severity. Moreover, the apparent harmony between color-coded biotype probes and mucosal thickness suggests their potential utility in clinical settings. Additionally, the analysis of MMP-8 and CLP biomarkers in PICF has been identified as an objective method for assessing peri-implant disease severity, with consistent findings between the two biomarkers. Secondarily, it was noted that the biotype did not alter the NSMT clinical parameters outcomes, and MMP-8 and CLP values decreased to a similar extent following NSMT, irrespective of biotype thickness.

## Data Availability

No datasets were generated or analysed during the current study.

## Declarations

The clinical trial was registered in the ClinicalTrials.gov database, first submitted on December 01, 2023 and published on December 15, 2023 (NCT06173739).
